# Integrated Multiparametric High-Content Profiling of Endothelial Cells

**DOI:** 10.1177/2472555218820848

**Published:** 2019-01-25

**Authors:** Erika Wiseman, Annj Zamuner, Zuming Tang, James Rogers, Sabrina Munir, Lucy Di Silvio, Davide Danovi, Lorenzo Veschini

**Affiliations:** 1 Stem Cell Hotel—Cell Phenotyping Platform, Centre for Stem Cells & Regenerative Medicine, King’s College London, London, UK; 2 Viadynamics, London, UK; 3 Tissue Engineering & Biophotonics, Dental Institute, King’s College London, London, UK; 4 Department of Industrial Engineering, Via Marzolo, Padua, Italy; 5 PerkinElmer (UK), Beaconsfield, UK

**Keywords:** endothelial cells, stem cells, iPS, phenotyping, high-content analysis

## Abstract

Endothelial cells (ECs) are widely heterogeneous at the cell level and serve different functions at the vessel and tissue levels. EC-forming colonies derived from induced pluripotent stem cells (iPSC-ECFCs) alongside models such as primary human umbilical vein ECs (HUVECs) are slowly becoming available for research with future applications in cell therapies, disease modeling, and drug discovery. We and others previously described high-content analysis approaches capturing unbiased morphology-based measurements coupled with immunofluorescence and used these for multidimensional reduction and population analysis. Here, we report a tailored workflow to characterize ECs. We acquire images at high resolution with high-magnification water-immersion objectives with Hoechst, vascular endothelial cadherin (VEC), and activated NOTCH staining. We hypothesize that via these key markers alone we would be able to distinguish and assess different EC populations. We used cell population software analysis to phenotype HUVECs and iPSC-ECFCs in the absence or presence of vascular endothelial growth factor (VEGF). To our knowledge, this study presents the first parallel quantitative high-content multiparametric profiling of EC models. Importantly, it highlights a simple strategy to benchmark ECs in different conditions and develop new approaches for biological research and translational applications for regenerative medicine.

## Introduction

**Figure 1.**
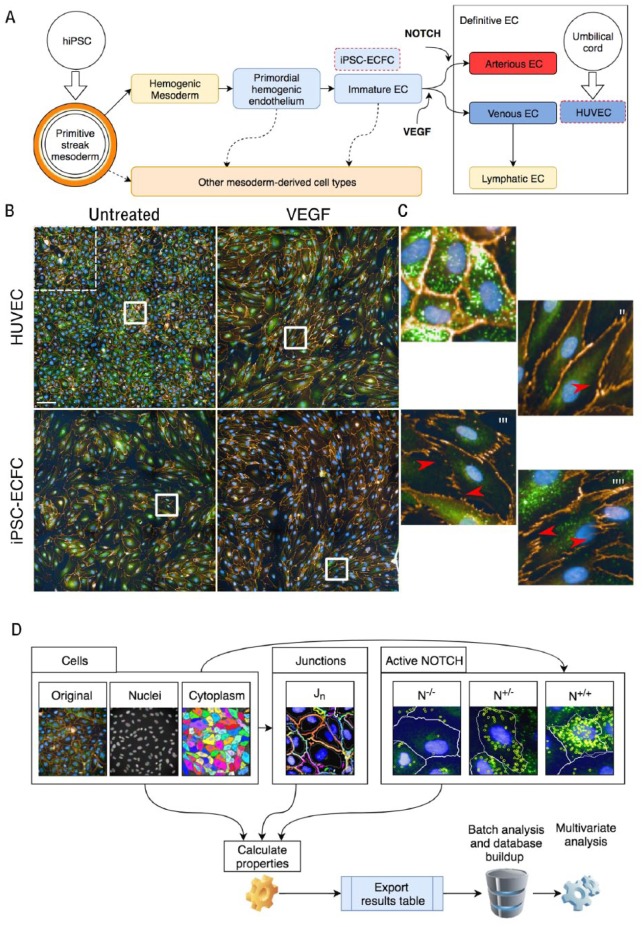
EC characterization using high-content analysis. (**A**) Schematic representation of the origin of the examined cell types for this study. HUVECs are primary cells derived from the umbilical cord that are venous ECs. The range of ECs that can be derived from iPSCs is wider and less defined. (**B**) Microphotographs comparing HUVECs and iPSC-ECFCs untreated and upon exposure to VEGF; tile from nine microscopic fields, one of which is highlighted in the dotted white square in the top left. (**C**) At higher magnification, panels i and ii refer to HUVECs untreated or treated with VEGF, respectively, and panels iii and iiii refer to iPSC-ECFCs untreated or treated with VEGF, respectively. Red arrows highlight discontinuation in junctions. (**D**) Schematic of workflow for image acquisition, quantification, and analysis (further details are available in the Supplemental Material) describing in particular the modules for cell morphology, junctions, and NOTCH, with sample images of the segmented objects.

Endothelial cells (ECs) form the inner lining of all blood and lymphatic vessels and are key mediators of functions such as vascular permeability, leukocyte trafficking, and angiogenesis (the formation of new blood vessels out of preexisting ones). Stable properties of specialized vessel types emerge from the collective behavior of neighboring heterogeneous ECs. Moreover, specific vasculature in tissues is often composed of functionally heterogeneous vessels (such as arteries, veins, lymphatics, and sinusoids). This diversity of function and complexity of scale (at the cell, vessel, and tissue level) are reflected in a remarkable degree of phenotypic heterogeneity. In essence, each of our ~6.2 × 10^11^ ECs (on average, in humans, ~22% of all nucleated cells and ~3% of all cells^1–3^) can be considered phenotypically distinct from all others (**Fig. 1A**).^4^

Experimentally, in vitro, this cell heterogeneity presents challenges for phenotypic characterization. Importantly, transformative changes are taking place in cell-based assays aimed at accurately profiling cells. For example, (1) complex cultures (3D, bioprinting, organ-on-a-chip) are surpassing more traditional 2D cultures; (2) unbiased analysis of morphological parameters in endpoint and/or dynamic imaging in live assays is complementing antibody-based cell markers; and (3) primary or induced pluripotent stem cell (iPSC)-derived cell lines capturing the genetic backgrounds of single individuals are becoming available. These shifts bring new exciting opportunities for translational research. Nonetheless, it is less often highlighted that these changes also present substantial challenges in the acquisition and analysis of data, requiring innovative workflows and new approaches to integration. Importantly, novel versus traditional cell systems are rarely compared quantitatively and side by side.

We have recently described novel analysis tools to “benchmark” cells accounting for interexperimental variation^5,6^ with a view of future applications to include dynamic imaging.^7^ Moving forward substantially from previous work around neural stem cells,^8^ these approaches were developed for iPSCs and other cell types^9^ and offer unprecedented possibilities to combine experiments from different conditions into single coherent datasets.

Monolayer sheets of ECs are widely employed as a model for stable endothelia. Cell phenotype is commonly evaluated via observation of immunofluorescence images.^10,11^ Human umbilical vein ECs (HUVECs) are the most diffuse model. Remarkably, to our knowledge, no unbiased morphology approach to compare iPSC-derived EC types with HUVECs has been described to date. In the context of tissue development and growth, ECs play a fundamental role in chaperoning/directing the formation of tissue functional units.^12^ Thus, obtaining tissue-specific iPSC-derived ECs (iPSC-ECs) to be used in microtissue engineering is an appealing goal. These models offer significant potential for precision medicine and may provide a route to autologous cell therapy. Currently, available protocols for iPSC-ECs mirror some of the heterogeneity found in vivo.^13^ Yet, stepwise procedures typically require the addition of specific growth factors at defined time points for more than 10 days. Cells are usually characterized via the expression of lineage markers at defined time points where intermediate populations are not analyzed extensively. Therefore, achieving “deep” characterization of iPSC-EC phenotypes individually, collectively, and temporally will lead to an improved understanding of the biology and definition of protocols with profound implications for research and translation.

High-content image analysis provides phenotypic information at the subcellular, single-cell, and population levels. For example, the abundance and spatial distribution of vascular endothelial cadherin (VEC) throughout the EC membrane offers key information regarding cell activation status, including propensity to migrate or form a stable quiescent monolayer.^14^ NOTCH signaling is a key driver of EC specialization and a regulator of interendothelial adhesiveness and EC junctional stability.^15^ Thus, combining the study of VEC distribution and NOTCH activation at the cell level in association with morphological parameters and context features can result in a wealth of information regarding EC regulatory status under specific conditions.

Here, we report a high-content EC phenotyping platform using morphology, VEC staining, and analysis of NOTCH activation. Cell spreading and elongation (in “migratory” phenotypes) are key features to dissect diverse stages of differentiation. We therefore defined a method to assess these in an unbiased manner with a high-content approach. We introduce morphology parameters (e.g., cell and nuclei area, roundness, width/length ratio together with an array of Symmetry, Threshold compactness, Axial or Radial [STAR] features; see Supplemental Material for a complete list). Importantly, subcellular analysis of features (with analysis of junctions), population stratification (via the NOTCH status), and context features (quantification of size for clusters of NOTCH-positive cells) are collated. As the HUVEC response to vascular endothelial growth factor (VEGF) is very well characterized,^10,14^ we used this cell system as a reference. Subsequently, we employed our validated pipeline to investigate the phenotype of unstimulated or VEGF-activated iPSC-EC-forming colonies (iPSC-ECFCs).^16^ Based on their derivation protocols,^16^ these cells can be considered fully committed endothelial progenitors rather than definitive, mature, fully specialized ECs.

Altogether, the workflow described here serves as a roadmap toward phenotyping of ECs from different sources. This will help in characterizing phenotypes of ECs under different experimental conditions. Methods like the one described will hereby support the development and quality control of protocols for iPSC differentiation toward specialized (arterial, venous, lymphatic) or tissue-specific (renal glomerulus, liver sinusoid, etc.) cells for translational applications. Furthermore, characterization of diverse EC populations would open new routes to target pharmacologically specific EC subpopulations in precision medicine.

## Materials and Methods

### Cell Culture Reagents

HUVECs and iPSC-ECFCs^16^ (PromoCell, Heidelberg, Germany and Axol Bioscience, Cambridge, UK, respectively) were plated on 10 µg/mL fibronectin (from human plasma, Promocell)-coated flasks, grown in EGM 2 medium (Promocell) in the absence of antibiotics, detached with Accutase (Thermo Fisher Scientific, Waltham, MA), and used by passage 3. For experiments, 5 × 10^4^ ECs were seeded in the center of 22 × 22 mm fibronectin-coated coverslips housed within a six-well plate well and cultured for 48 h under basal (EGM 1, Promocell) or activated (EGM 1 + 50 ng/mL VEGFA, Peprotech, London, UK) conditions in duplicate. EGM 1 medium containing 1 ng/mL basic fibroblast growth factor (bFGF), 100 pg/mL EGF, and no VEGF was chosen in order to maintain the cells in quiescent conditions. The ECs formed confluent monolayers at the center of the slide where images were acquired.

### Immunostaining

Slides were fixed with 2% para-formaldehyde in phosphate-buffered saline (PBS) for 10 minutes and then washed extensively with PBS supplemented with 1% fetal bovine serum (FBS) and incubated (45 minutes) with Alexa 594-conjugated antibody against VEC (1:200, 1 µg/mL, BioLegend, San Diego, CA). VEC is a lineage marker for ECs and cells were all VEC+ (see **Fig. 1B**). After permeabilization (1 minutes) in 0.1% Triton X-100, cells were incubated (45 minutes) with primary anti-activated NOTCH antibody (Abcam 1:200, 1 µg/mL final). Subsequently, plates were washed and incubated with 1 µg/m: secondary Alexa 488-conjugated antibody (30 minutes, Thermo). Nuclei were stained with Hoechst 33342 (1 µg/mL, 10 minutes, Sigma) and slides were mounted with Mowiol (Sigma).

### Image Acquisition and Analysis

We obtained images from slides with an Operetta CLS system (PerkinElmer, Waltham, MA) equipped with a 40× water-immersion lens (Numerical Aperture 1.1). On each slide, five areas were acquired. Each area is composed of nine microscopic fields at 40× magnification tiled with 0.05% overlapping (**Fig. 1B**). For EC characterization, we designed the modular pipeline (represented schematically in **Figure 1C** and detailed in Supplemental Figures). We collected a total of 47 features derived from the modules described in the Supplemental Material. Briefly, we first identified nuclei with Hoechst and cytoplasm using the VEC marker (Ch A555, orange) for each cell and measured parameters including cell/nuclei area, length, and roundness. We subsequently identified the junctional area as peaks of VEC stain and assigned each object to its respective cell. We defined “junctions” as regions in the image that give a strong signal in the VEC marker and generate “edges” (according to the SER edge algorithm in the pipeline; see Supplemental Material). We calculated the average number of VEC-positive objects per nuclei (J_n_). This novel method was inspired by a previously reported study classifying EC junctions based on VEC staining.^15^ In our method, high J_n_ corresponds to “active” junctions while low J_n_ refers to “inhibited” or “stable” junctions, which have been shown to correlate consistently with NOTCH signaling. Finally, we identified activated NOTCH as bright cytoplasmic or nuclear spots. We assigned each cell to N^–/–^ (cell without spots, not active), N^+/–^ (cell with spots in the cytoplasm only, not transcriptionally active), or N^+/+^ (cell with spots in cytoplasm and nucleus or nucleus alone, putatively active). No significant number of cells were identified with spots in the nucleus only, and therefore these were grouped in the N^++^ category. We divided cells in these groups based on current knowledge regarding NOTCH protein compartmentalization during NOTCH pathway activation.^17^ The antibody used in our assay reveals “activated NOTCH,” which corresponds to cleaved NOTCH intracellular domain (NICD). As a context feature, we evaluated the number of N^+/+^ cells in contact with each other (NOTCH clusters; see **Fig. 1** and Supplemental Material); the clustering method was adapted from previous work in our laboratory.^6,18^ We ran the pipeline described using Columbus software (PerkinElmer) on a virtual machine in batch. Images for each experimental condition in duplicate (40 images composed of nine tiles at 40× OM in total) were analyzed and numerical data exported. .txt files were directly imported into Spotfire (Tibco Software, Palo Alto, CA) through the Columbus data repository link.

### Data Integration and Analysis

Data were tagged prior to running High Content Profiler (HCP) in Spotfire (Tibco): cell type (HUVEC vs iPSC-EC), treatment (untreated vs VEGF). HCP was launched with the following settings: Other screen—plate well based—well analysis results—select all features (excluding metadata)—select relevant annotations—run HCP. Different data exploration tabs were generated. Features overview was used to capture feature values across plates (e.g., mean percentage of cells in the NOTCH categories) using raw data. We used hierarchical cluster analysis to identify clusters in an unsupervised fashion. Column data were classified by cell type and treatment, and distances between features were measured according to Euclidean distance, normalized by mean and weight ordered by average value. For exploration of data structure, a principal component analysis (PCA)-generated distribution was visualized with cell type + treatment. Statistical analysis of relevant parameters was performed with GraphPad Prism. We performed one- and two-way ANOVA (as appropriate) followed by post hoc test for multiple comparisons to assess statistical significance.

## Results

### HUVECs in the Absence or Presence of VEGF Reveal Changes in Phenotypic Features

**Figure 2.**
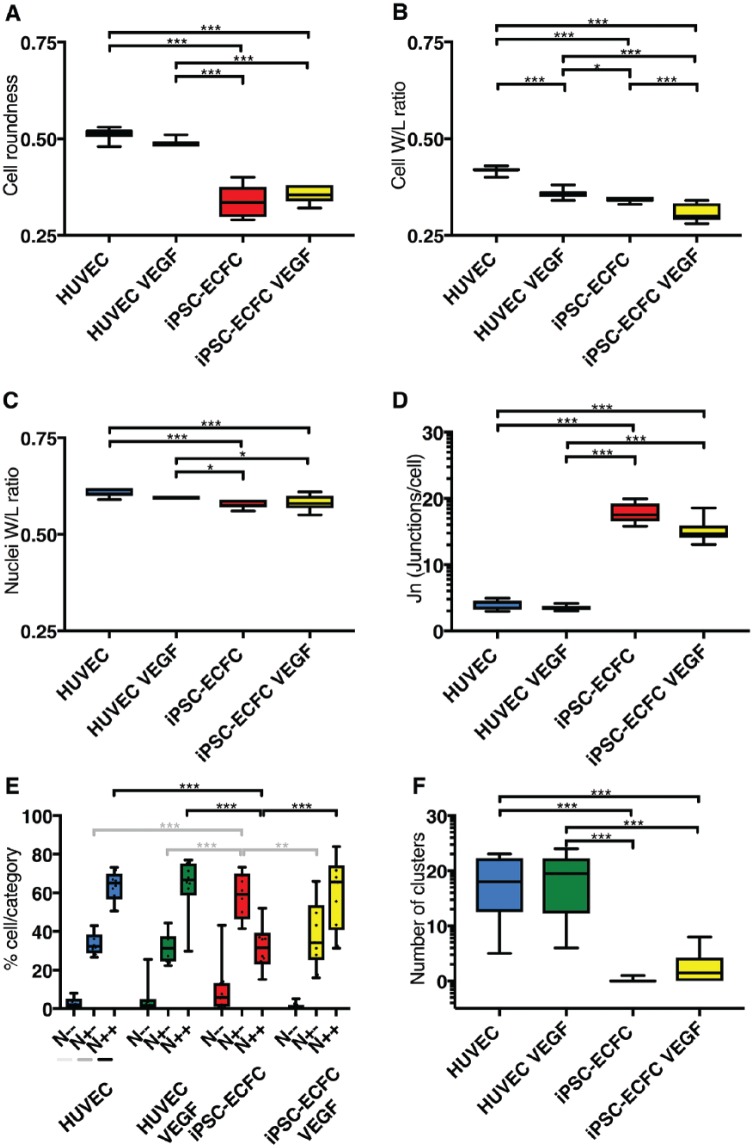
Selected features: morphology, junctions, NOTCH. HUVECs and iPSC-ECFCs in the absence and presence of VEGF are analyzed for cell morphology features such as roundness (**A**) and width-to-length ratio (**B**). Differences between cell types are apparent, and the cell width-to-length ratio is significantly changed in response to VEGF, whereas the nuclear width-to-length ratio (**C**) is not. (**D**) Quantification of J_n_ shows differences between the cell types. (**E**) NOTCH activation pattern for each experimental condition reveals a response of iPSC-ECFCs to VEGF. Statistical analysis, with ANOVA *p* values as follows: *p < 0.05, **p < 0.01, ***p < 0.001.

Confluent EC monolayers are widely used to model the endothelial barrier.^19,20^ In order to test our imaging workflow strategy and validate our approach, we first set out to evaluate HUVECs, a well-established EC model.^10,19^ HUVECs under basal (quiescent) conditions demonstrate a polygonal shape with tight and continuous interendothelial junctions. Staining HUVECs with Hoechst and VEC highlighted classic cobblestone-like morphology (**Fig. 1B**). Under these conditions, ECs appeared small and in contact with each other, forming a continuous barrier. As expected,^21^ upon VEGF treatment, cells changed in shape, becoming stretched and elongated (**Fig. 1B**). We therefore hypothesized that VEGF treatment would elicit in HUVECs changes in morphology features that could be quantified by our image analysis pipeline. The ratio between cell width and length varied significantly, as cells were more stretched upon VEGF activation (**Fig. 2B**). Cell roundness, nuclei roundness, and nuclei width-to-length ratio (**Fig. 2A,C,D**) were not significantly affected by the presence of VEGF. This change was consistent with a stretched “activated” cell morphology.

In microscopic images, we observed that VEC-stained junctions appeared discontinuous, interdigitated, and jagged (**Fig. 1B**). In our pipeline, we identified discrete VEC-stained regions surrounding each cell. We refined a parameter (J_n_; see Materials and Methods and Supplemental Material) measuring the number of junctional objects per cell. We reasoned that J_n_ could be used as a proxy for the continuity of junctions and may increase in cells with jagged junctions, as these present areas where the signal is much weaker (**Fig. 1C**, arrowhead). No significant difference for J_n_ was reported in HUVECs cultured in the absence or presence of VEGF (**Fig. 2D**).

Activated-NOTCH dots were visible in microscopic images (**Fig. 1B**; see Supplemental Material). Nonetheless, via simple observation, no clear-cut obvious difference in activated-NOTCH stain could be observed upon VEGF treatment as patterns appeared virtually undistinguishable from untreated conditions and differences were difficult to quantify (**Fig. 1B**). We then set out to quantify NOTCH activation using our automated pipeline. HUVECs had a high baseline NOTCH activity (>20% and >60% in the N^+/–^ and N^+/+^ categories, respectively) and VEGF treatment did not affect this distribution (**Fig. 2E**). The size of NOTCH-positive cell clusters presented a slight, not significant, increase upon VEGF treatment (**Fig. 2F**).

Overall, our observation and measurements are consistent with an “activation” effect of VEGF to the endothelium in HUVECs as seen by changes in the width/length ratio. Nevertheless, no major change was observed in J_n_ and NOTCH in HUVECs upon VEGF treatment, consistent with the possibility of some level of basal activation.

### iPSC-EC Reveal a Distinct Phenotype to HUVECs, Confirmed by Unsupervised Clustering

HUVEC is a widely used and well-established model that arguably presents several limitations.^20^ ECs derived from iPSCs (iPSC-ECs) are considered more relevant models to study ECs. For example, it is possible to obtain a wider range of specialized cell types other than large-vein ECs. We therefore set out to observe HUVECs and iPSC-ECFCs in the absence or presence of VEGF.

Microscopic images (**Fig. 1B**) showed that untreated iPSC-ECFCs appeared distinct from HUVECs. The quantification of morphological features (**Fig. 2A–D**) showed a higher variance of the measured parameters, indicating a more phenotypically diverse cell population. In some cases, iPSC-ECFCs were more similar to VEGF-treated HUVECs (cell width/length ratio, **Fig. 2B**). Junctions appeared very different in microscopic images (**Fig. 1B**), and J_n_ was significantly higher in iPSC-ECFCs (**Fig. 2D**) and responsive to VEGF. These results were consistent with looser intercellular junctions in iPSC-ECFCs.

We later set out to quantify the response of iPSC-ECFCs to VEGF in terms of NOTCH activation. Untreated iPSC-ECFCs were significantly more abundant in the N^+/–^ and less abundant in the N^+/+^ category compared with HUVEC (**Fig. 2E**). Importantly, whereas VEGF had no observable effect on HUVECs, VEGF induced a significant increase in the N^+/+^ category and a decrease in the N^+/–^ category in iPSC-ECFCs. Altogether, these results validated the selected feature changes observed in microscopic images, suggesting that iPSC-ECFCs present a more activated phenotype than HUVECs and a differential response to VEGF.

We hypothesized that cell types (HUVECs vs iPSC-ECFCs) would be diverse enough and the phenotypic features acquired would be sufficient to distinguish these cell populations. In other words, in our experimental conditions we could run unsupervised clustering, capturing, in an unbiased manner, object populations reflective of diverse cell behavior. To test our hypothesis, we performed multidimensional reduction and visualization. PCA for the three principal components reported an explained variance of more than 80%. The variance explained with principal component 1 was 54% and rose to 74% with component 2 and 81% with component 3 (Supplemental Material).

**Figure 3.**
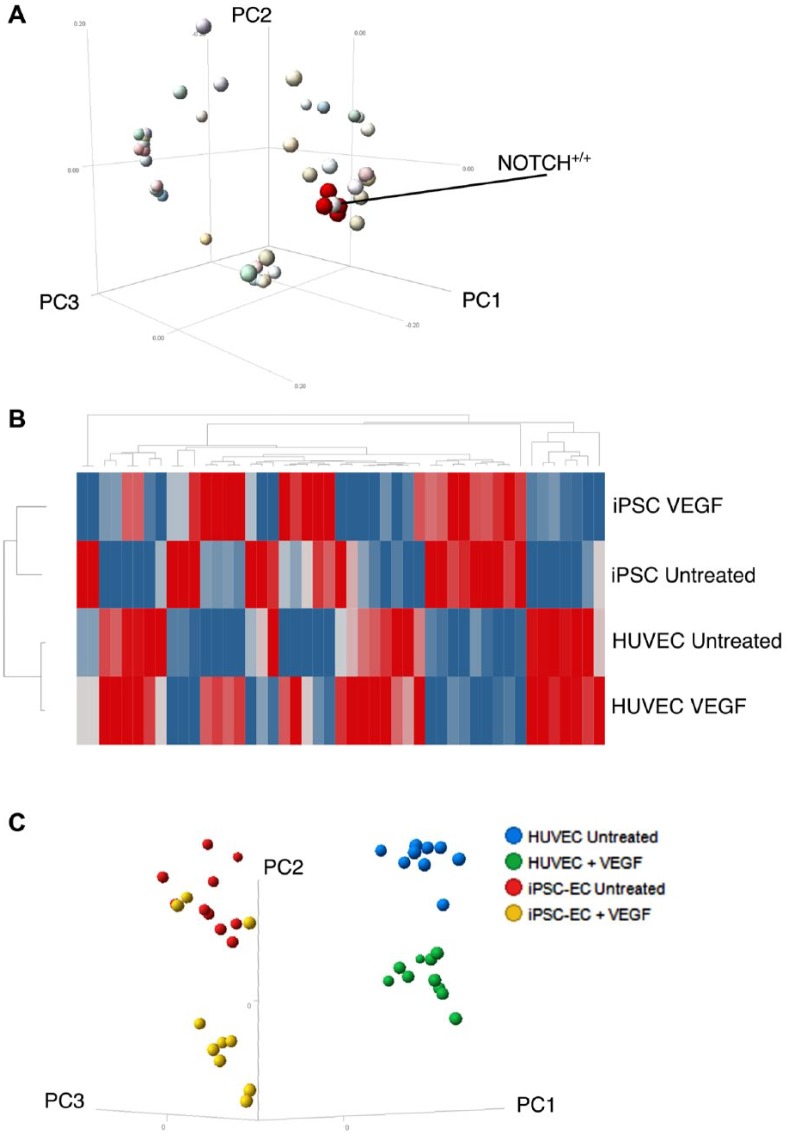
Multidimensional reduction. (**A**) The obtained 47 features analyzed are projected in terms of their loadings on the three principal components. Note that four features referring to the NOTCH cluster size (in red) neighbor the N^+/+^ NOTCH activation category percentage. (**B**) Hierarchical clustering of the four conditions. (**C**) PCA reveals separation for HUVECs in the absence or presence of VEGF in a distinct cluster to iPSC-ECFCs.

We observed the loading of 47 features (see Supplemental Material), including all of those described above into the first three principal components (**Fig. 3A**). All four NOTCH cluster parameters (**Fig. 3A**, red dots) loaded in a very similar way to the PCA, as expected. Also expectedly, the N^+/+^ category percentage value loaded in a neighboring way (**Fig. 3A**, highlighted). Other STAR and morphological features were surrounding this group of NOTCH-related features, suggesting that these could be predictive of NOTCH status in this setting.

In hierarchical clustering, different populations formed discrete clusters demonstrating that our analysis can separate cell types (**Fig. 3B**). This was apparent in a PCA plot (**Fig. 3C**). Altogether, these results provide a defined set of parameters to clearly distinguish different EC populations.

## Discussion

Previous studies have highlighted the feasibility of using in vitro high-content analysis with ECs to model aspects of angiogenic signaling and microvessel formation. Tubular network formation was evaluated in the HUVEC/fibroblast (HDF) co-culture assay.^23^ In other cases, multiparametric phenotypic profiles generated from a bespoke informatics platform were applied to screen compounds. High-throughput assays focused on vascular assembly incorporated image-derived information from HUVEC nuclei.^23,24^

Moreover, comparisons between different EC types (primary and iPSC derived) have recently been reported with image analysis with respect to sprouting angiogenesis. The authors pointed to an impaired angiogenic potential of iPSC-ECFCs, possibly due to a less mature phenotype.^25^

Finally, the importance of VEC and NOTCH signaling cross-talks has been stressed.^15,26,27^ Altogether, these considerations prompted us to quantify phenotypic features and use multiparametric high-content analysis with the precise goal of profiling distinct cellular phenotypes.

In this study, we offer a guide toward unbiased characterization of ECs using multidimensional reduction of multiparametric high-content analysis data. We have sought to design a framework for EC phenotyping to evaluate cell morphology and two markers (VEC and active NOTCH) indicative of EC activation. Qualitative observation suggested that iPSC-ECs used in this study were morphologically different from HUVECs, suggesting a differential activation status at baseline. Dissection of the molecular determinants of these EC phenotypes is beyond the scope of the present work.

Since HUVECs responded to VEGF in shape and not in J_n_ and NOTCH readouts, these parameters were useful to elicit differences between the cell types. We here describe a workflow to analyze distinct EC models in different conditions. The high-resolution images we obtained are comparable in quality and magnification to those from studies aimed at characterizing ECs that do not refer to high-content-based methods for analysis.^10^ We stained nuclei and evaluated phenotypic features from objects, cell–cell interactions (junctions), and subcellular NOTCH staining (including context features).

HUVECs are an established cellular model for the study of vascular biology and angiogenesis. They have been the key to several findings.^22^ HUVECs are nonetheless a cell type derived from a specific body location (umbilical cord) and should be considered an effective model of ECs specified toward large-vein fate.^11^ Qualitative evaluation confirmed previous published observations where VEGF treatment induced prototypical morphological changes in HUVECs. It is interesting to observe, though, that the differences appear significant for ratio width/length but not for roundness, suggesting a more “stellate” cell shape, which has been proposed to be associated with an activated migratory environment-probing^28^ phenotype. It is also interesting that the nuclear width/length (**Fig. 2C**) does not appear to be a good proxy for this phenotype. Many mechanisms of EC biology have been investigated in HUVECs, and thus understanding the extent to which it is possible to compare novel cell types such as iPSC-ECFCs side by side is of significant importance.

New possibilities are arising thanks to the development of iPSC technology. IPSC-ECs can be derived following protocols developed in recent years, and these protocols could enable us to produce specific cell types resembling the ample range of ECs found in vivo. Advantages are offered by iPSC-ECs, as cells can be derived from specific individuals for precision medicine and regenerative medicine, and importantly, the type of cells derived could be broader. Consistently, a wider range of feature variance was observed in this study for iPSC-ECFCs with respect to HUVECs, including the response to VEGF. Overall, these results demonstrate that iPSC-ECFCs and HUVECs model distinct EC types and suggest that iPSC-ECFCs have a more heterogeneous/plastic phenotype.

Our measurements showed a clear difference in J_n_ in cells with obviously discontinuous junctions but failed to resolve finer differences between untreated and VEGF-treated HUVECs (**Figs. 1B and 2D**). We conclude that J_n_ is currently a good proxy to discriminate cells with continuous or discontinuous junctions but not finely tuned to detect subtler differences in HUVECs in the absence or presence of VEGF (see arrow in **Fig. 2B**). Further improvement and complementary measures, including machine learning-based classification, could be deployed to classify cells presenting linear versus interdigitated junctions.

NOTCH activation analysis revealed a difference in the distribution of cells into the three categories. The iPSC-ECFCs had a significant fraction (with variance) in the N^–/–^ category, while HUVECs were more abundant in the N^+/–^ category and when untreated presented a consistent fraction of N^+/+^. This strongly suggests that iPSC-ECFCs present an intrinsically lower degree of basal activated NOTCH signaling, where this may be already maximally activated in HUVECs. NOTCH activation or other signaling readouts could in the future be predicted by morphological features that show a neighboring loading into multidimensional reduction components (**Fig. 3A**; see also Christiansen et al.^29^). Finally, NOTCH signaling, like many other signaling pathways, is extremely dynamic, and inclusion of time-lapse imaging could significantly enrich the content, providing new avenues to resolve differences and characterize cell model systems.

The framework provided could in fact be extended to dynamic imaging data and other diverse biological datasets. For example, molecular characterization techniques such as Western blotting, quantitative real-time PCR, and RNA sequencing, which provide information at the population level, may be integrated downstream if required. In the near future, these approaches will be attempted more and more across different experiments and across different laboratories to allow the analysis of variation to overall increase experimental data reproducibility. Multivariate analysis allows the stratification of cell populations and conditions throughout multiple experiments. Importantly, predictions of correlations between different parameters emerge, allowing serial analyses (e.g., different multicolor panels as with cytofluorimetric analysis) on the same cell populations or in titration experiments. This could significantly improve the development of robust protocols for iPSC differentiation. Moreover, such strategies can easily be extended to other relevant markers from the same experimental conditions (in multiwell plates) and data can be integrated in a single database.

Important changes are taking place in cell-based assays that extend from the current limitations of traditional cell cultures to explore more complex environments.^30^ Novel cell models have been proposed, with particular emphasis on 3D culture systems and dynamic analysis of live image data. These systems render assay development, data collection, and analysis workflows more complex. Irrespective of the problems of mere computational power, a major bottleneck for full fruition is integration of the data. Quantitative comparison of different cell models can then be applied to diverse cell systems. For selected cell types such as ECs, it is tempting to speculate that it would be more fruitful to build agile data-integrated analysis platforms first in 2D, as these cells form subtle endothelia in vivo, which may well be mirrored in these conditions. Solutions, including some highlighted here, may next be adapted to complex 3D cultures.

## Supplemental Material

DS_DSIC820848 – Supplemental material for Integrated Multiparametric High-Content Profiling of Endothelial Cells Click here for additional data file. Supplemental material, DS_DSIC820848 for Integrated Multiparametric High-Content Profiling of Endothelial Cells by Erika Wiseman, Annj Zamuner, Zuming Tang, James Rogers, Sabrina Munir, Lucy Di Silvio, Davide Danovi and Lorenzo Veschini in SLAS Discovery
