# Supplementary material for: Integrated Multiparametric High-Content Profiling of Endothelial Cells
Source: SLAS Discov. 2019 Jan 25;24(3):264–73. doi: 10.1177/2472555218820848 (PMC6484530; doi:10.1177/2472555218820848)
Supplement: DS_DSIC820848 – Supplemental material for Integrated Multiparametric High-Content Profiling of Endothelial Cells [file DS_DSIC820848.pdf]

Supplemental material

Integrated multi-parametrical high-content profiling of endothelial cells

Erika Wiseman[1,2], Annj Zamuner[3,4], Zuming Tang[1,5], James Rogers[3], Sabrina Munir[1], Lucy Di Silvio[3], Davide Danovi[1,*], Lorenzo Veschini[3,*]

1 Stem Cell Hotel - Cell Phenotyping Platform, Centre for Stem Cells & Regenerative Medicine, King's College London, SE1 9RT, London, United Kingdom
2 Viadynamics, 52-54 Rosebery Avenue, EC1R 4RP, London, United Kingdom
3 Tissue Engineering & Biophotonics, Dental Institute, King's College London, SE1 9RT, London, United Kingdom
4 Department of Industrial Engineering, Via Marzolo, 35131, Padua, Italy
5 PerkinElmer (UK), Chalfont Road, Beaconsfield HP9 2FX, United Kingdom
*correspondence to: **lorenzo.veschini@kcl.ac.uk** and **davide.danovi@kcl.ac.uk**

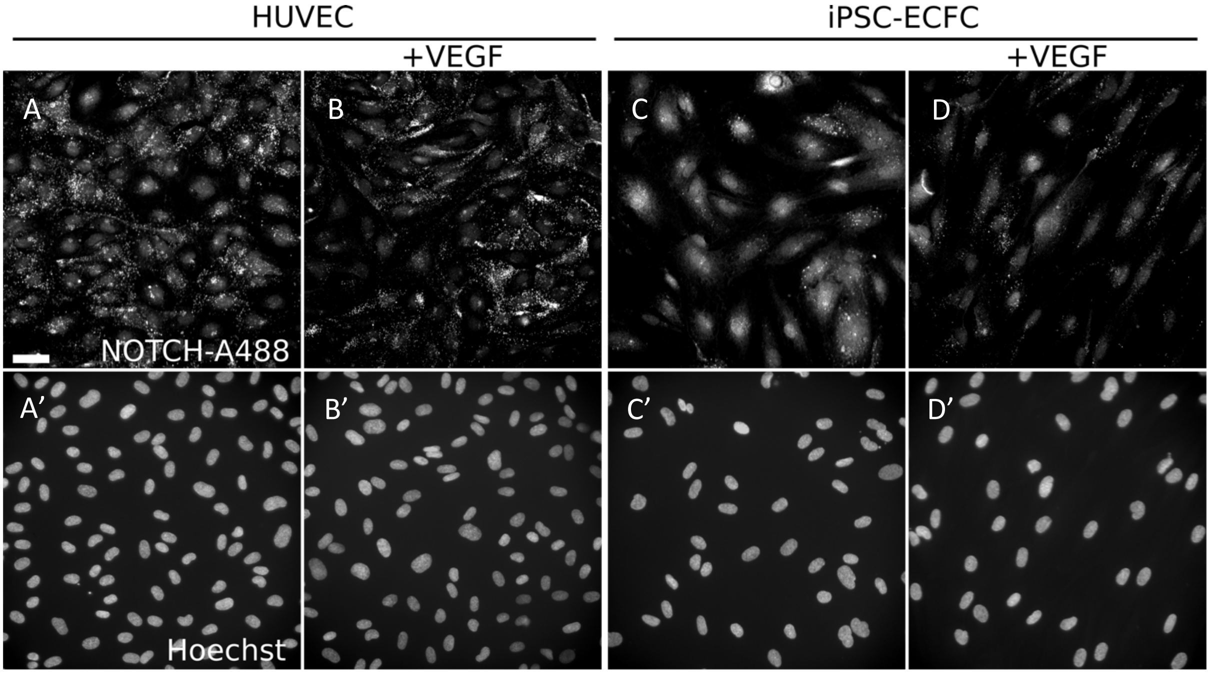

Figure S1: Representative images of individual NOTCH-A488 (A-D) and Hoechst (A'-D') channels for single microscopic fields (40X) under indicated experimental conditions. Scale bar = 40 μm.

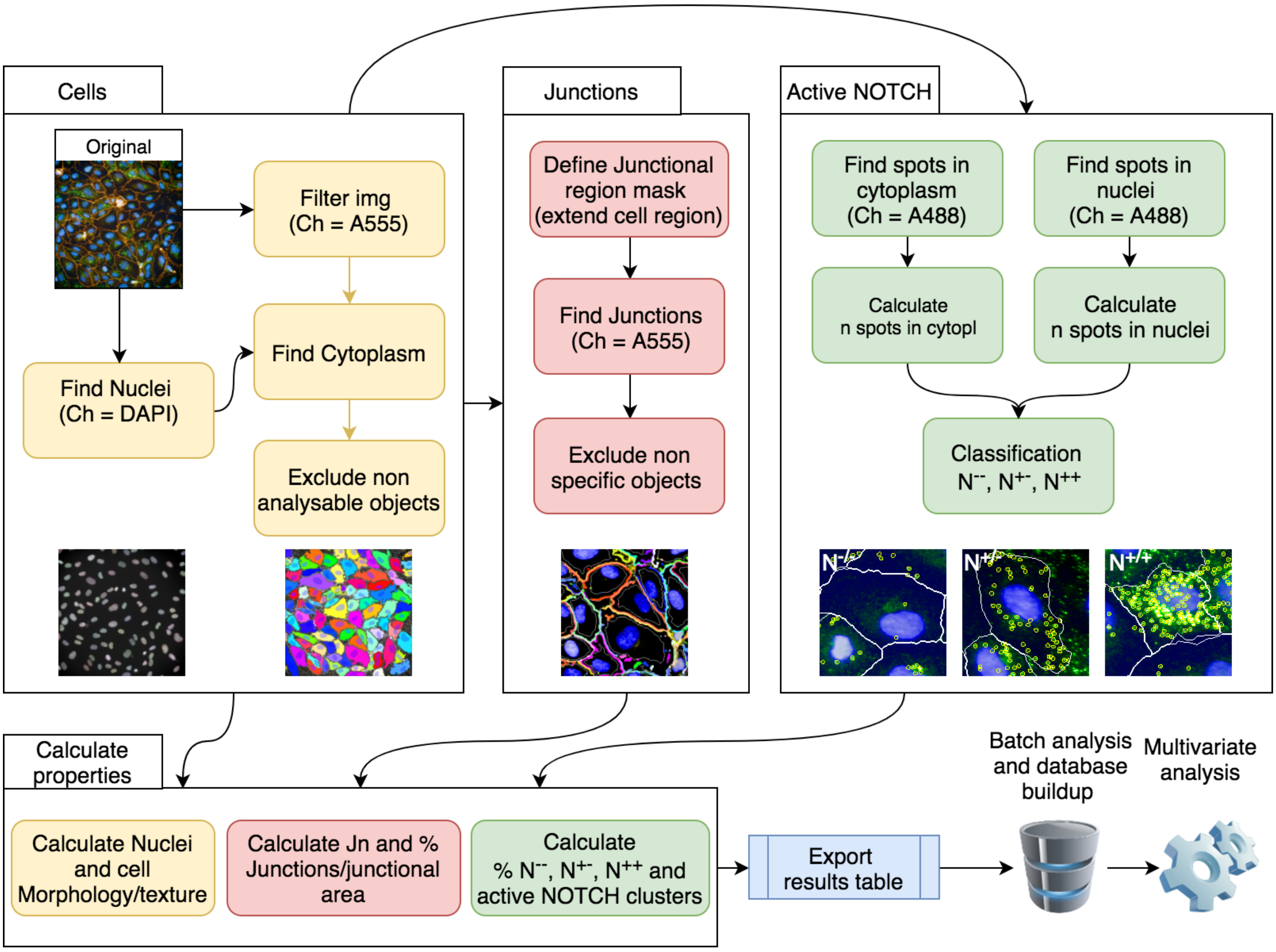

Figure S2: Schematics detailing the different steps to identify and measure phenotypic features (see Figure 1C and Table S1).

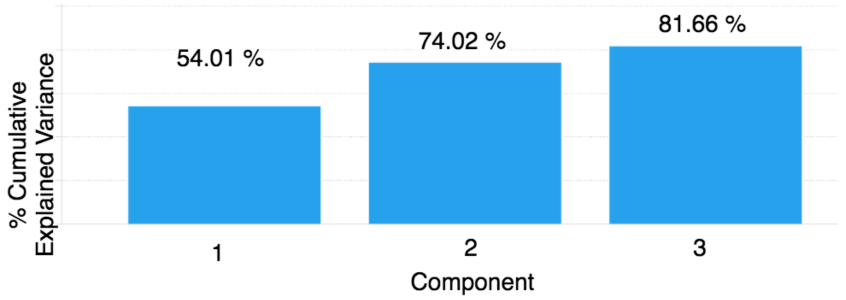

Figure S3: Cumulative explained variance for each Principal Component (PC). The first component (PC1) explain 54.01% of the total variance value which raises to 74.02% with PC2 and 81.66% with PC3.

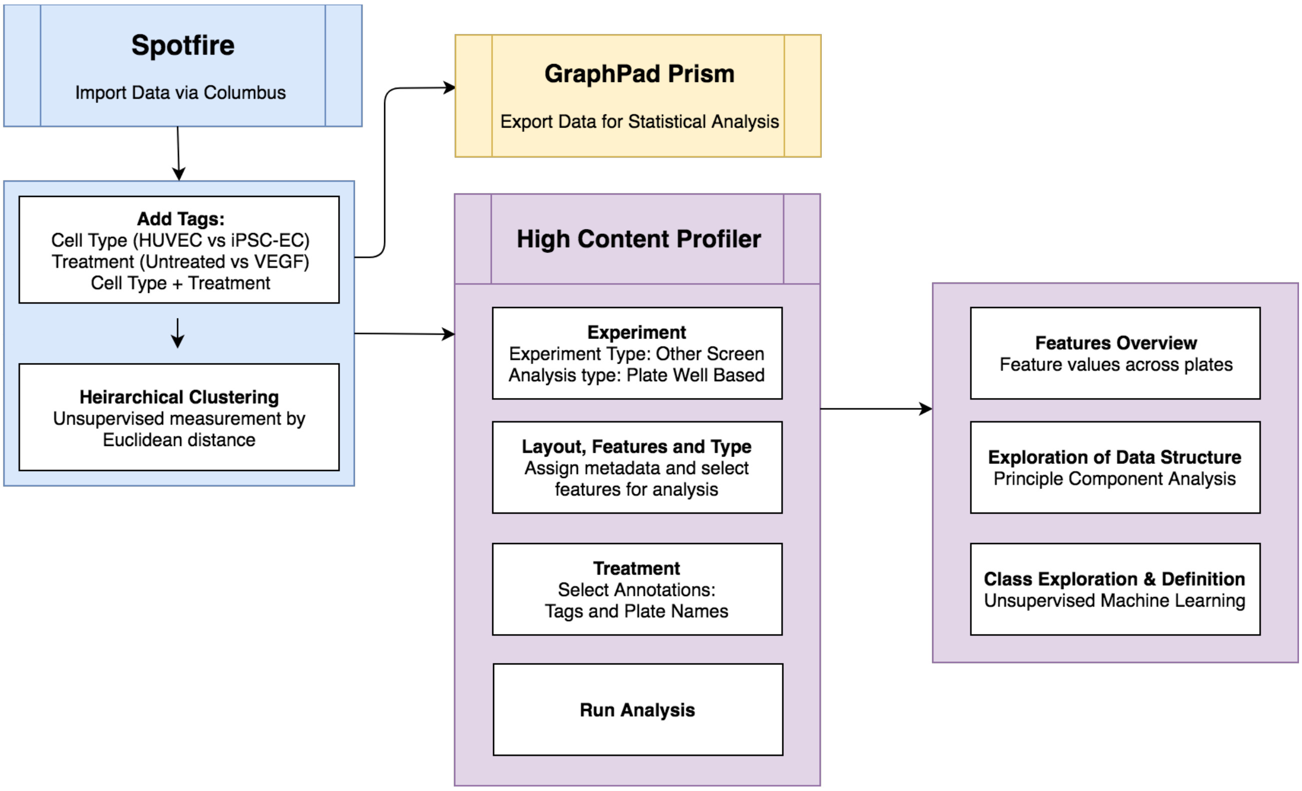

Figure S4: Flowchart representing the analytical post processing of data generated by the image analysis pipeline. Numerical data are collated in a single Tibco Spotfire database, tagged as appropriate and transferred to Graph Pad Prism for statistical analysis of variance (ANOVA) and to High Content Profiler for dimensionality reduction, clustering and PCA.

Number of Objects
% n +/+
% n-/-
% n+/-
Jn (% n Junctions final/n nuclei= avg Jn/cell)
NOTCH Clusters - Number of High Notch- N of High NOTCH per Cluster - Mean per Well
NOTCH Clusters - Number of Objects
NOTCH Clusters (>3 cells/cluster) - N of High NOTCH per Cluster - Mean per Well
NOTCH Clusters (>3 cells/cluster) - Number of Objects
Nuclei - Number of Objects
Cell Morphology STAR Axial Length Ratio - Mean per Well
Cell Morphology STAR Axial Small Length - Mean per Well
Cell Morphology STAR Profile 1/5 - Mean per Well
Cell Morphology STAR Profile 2/5 - Mean per Well
Cell Morphology STAR Profile 3/5 - Mean per Well
Cell Morphology STAR Profile 4/5 - Mean per Well
Cell Morphology STAR Profile 5/5 - Mean per Well
Cell Morphology STAR Radial Mean - Mean per Well
Cell Morphology STAR Radial Relative Deviation - Mean per Well
Cell Morphology STAR Symmetry 02 - Mean per Well
Cell Morphology STAR Symmetry 03 - Mean per Well
Cell Morphology STAR Symmetry 04 - Mean per Well
Cell Morphology STAR Symmetry 05 - Mean per Well
Cell Morphology STAR Symmetry 12 - Mean per Well
Cell Morphology STAR Symmetry 13 - Mean per Well
Cell Morphology STAR Symmetry 14 - Mean per Well
Cell Morphology STAR Symmetry 15 - Mean per Well
Cell Morphology STAR Threshold Compactness 30% - Mean per Well
Cell Morphology STAR Threshold Compactness 40% - Mean per Well
Cell Morphology STAR Threshold Compactness 50% - Mean per Well
Cell Morphology STAR Threshold Compactness 60% - Mean per Well
Cell Morphology Width [μm] (2) - Mean per Well
Nuclei Morphology Area [μm^2] - Mean per Well
Nuclei Morphology Length [μm] - Mean per Well
Nuclei Morphology Ratio Width to Length - Mean per Well
Nuclei Morphology Roundness - Mean per Well
Nuclei Morphology Width [μm] - Mean per Well
Cell Morphology Area [μm^2] - Mean per Well
Cell Morphology Length [μm] - Mean per Well
Cell Morphology Ratio Width to Length - Mean per Well
Cell Morphology Roundness - Mean per Well
[-/-] Cells having no spots - Mean per Well
[+/-] Cells having spots only in cytoplasm (no nuclei spots) - Mean per Well
[+/+] Cells having spots in both cytoplasm & nulcei - Mean per Well
[-/-] Cells having no spots - Number of Objects
[+/-] Cells having spots only in cytoplasm (no nuclei spots) - Number of Objects
[+/+] Cells having spots in both cytoplasm & nulcei - Number of Objects

Table S1: List of the 47 phenotypic features measured in our pipeline. Features highlighted in red correspond to the red dots in Figure 3 A.

**Input Image**	**Stack Processing :**<br>Maximum Projection<br>Flatfield Correction : None	
**Find Nuclei**<br>Output Population : Nuclei	**Channel :** DAPI **ROI :** None	**Method :** B<br>Common Threshold : 0.4<br>Area : > 30 µm2<br>Split Factor : 7.0<br>Individual Threshold : 0.4<br>Contrast : > 0.1
**Filter Image**<br>Output Image : Sliding Parabola	**Channel :** Alexa 555	**Method :** Sliding Parabola<br>Curvature : 4
**Filter Image**<br>Output Image : VEC SER Edge	**Channel :** Sliding Parabola	**Method :** Texture SER<br>Filter : SER Edge<br>Scale : 3 px<br>Normalization by : Kernel
**Filter Image**<br>Output Image : Inverted VEC SER Edge	**Channel :** VEC SER Edge	**Method :** Invert Image<br>Cut-off Quantile : 90
**Find Cytoplasm**	**Channel :** Inverted VEC SER Edge<br>**Nuclei :** Nuclei	**Method :** A<br>Individual Threshold : 0.25
**Calculate Morphology Properties**<br>Output Properties : Cell Morphology	**Population :** Nuclei<br>**Region :** Cell	**Method :** Standard<br>Area<br>Roundness<br>Width<br>Length<br>Ratio Width to Length
**Select Population**<br>Output Population : Nuclei Selected	**Population :** Nuclei	**Method :** Common Filters<br>Remove Border Objects<br>Region : Cell
**Select Population**<br>Output Population : Nuclei Selected Final	**Population :** Nuclei Selected	**Method :** Filter by Property<br>Cell morphology Area [µm2] : > 200
**Calculate Morphology Properties**<br>Output Properties : Nuclei Morphology	**Population :** Nuclei Selected Final<br>**Region :** Nucleus	**Method :** Standard<br>Area<br>Roundness<br>Width<br>Length<br>Ratio Width to Length
**Calculate Morphology Properties**<br>Output Properties : Cell Morphology	**Population :** Nuclei Selected Final<br>**Region :** Cell	**Method :** Standard<br>Area<br>Roundness<br>Width<br>Length<br>Ratio Width to Length
**Calculate Morphology Properties**<br>Output Properties : Cell Morphology STAR	**Population :** Nuclei Selected Final<br>**Region :** Cell	**Method :** Standard<br>Channel : Alexa 488<br>Symmetry<br>Threshold Compactness<br>Axial<br>Radial<br>Profile<br>Profile Inner Region : Nucleus<br>Profile Width : 4 px<br>**Sliding Parabola**<br>Curvature : 10
**Filter Image**<br>Output Image : VEC SER Ridge	**Channel :** Alexa 555	**Method :** Texture SER<br>Filter : SER Ridge<br>Scale : 5 px<br>Normalization by : Region Intensity
**Find Image**<br>Output Population : Junctions Points<br>Output Region : Junctions Region	**Channel :** VEC SER Ridge<br>**ROI :** None	**Method :** Common Threshold<br>Threshold : 0.8<br>Split into Objects<br>Area : > 200 px2
**Select Region**<br>Output Region : Junctions Region Selected	**Population :** Junctions points<br>**Region :** Junctions region	**Method :** Restrict by Mask<br>Population : Nuclei<br>Mask Region : Nucleus<br>Use Inverted Mask
**Modify Population**<br>Output Population : Number of Junctions final<br>Output Region : Selected Junctional region	**Population :** Junctions Points<br>**Region :** Junctions Region Selected	**Method :** Cluster by Distance<br>Distance : 0 px<br>Area : > 150 px2
**Find Spots**<br>Output Population : Spots in cytoplasm selected	**Channel :** Alexa 488<br>**ROI :** Nuclei Selected final / Cytoplasm	**Method :** C<br>Radius : ≤ 7 px<br>Contrast : > 0.15<br>Uncorrected Spot to Region<br>Intensity : > 1<br>Distance : ≥ 3 px<br>Spot Peak Radius : 0.8 px<br>Calculate Spot Properties

**Calculate Properties**	**Population :** Nuclei Selected final	**Method :** By Related Population
Output Properties : per Cell		Related Population : Spots in cytoplasm selected
		Number of Spots in cytoplasm selected
**Find Spots**	**Channel :** Alexa 488	
Output Population : Spots in cytoplasm selected	**ROI :** Nuclei Selected final / Nuclei	**Method :** C
		Radius : ≤ 7 px
		Contrast : > 0.15
		Uncorrected Spot to Region
		Intensity : > 1
		Distance : ≥ 3 px
		Spot Peak Radius : 0.8 px
		Calculate Spot Properties
**Calculate Properties**	**Population :** Nuclei Selected final	**Method :** By Related Population
Output Properties : per Cell		Related Population : Spots in cytoplasm selected
		Number of Spots in nuclei selected
**Select Population**	**Population :** Nuclei Selected final	**Method :** Filter by Property
Output Population : [-/-] Cells having no spots		F1 : Number of Spots in cytoplasm selected- per Cell :<3
		F2 : Number of Spots in nuclei selected- per Cell : < 3
		Boolean Operations : F1 and F2
**Select Population**	**Population :** Nuclei Selected final	**Method :** Filter by Property
Output Population : [+/-] Cells having spots		F1 : Number of Spots in cytoplasm selected- per Cell :>=3
		F2 : Number of Spots in nuclei selected- per Cell : < 3
		Boolean Operations : F1 and F2
**Select Population**	**Population :** Nuclei Selected final	**Method :** Filter by Property
Output Population : [+/+] Cells having spots		F1 : Number of Spots in cytoplasm selected- per Cell :>=3
		F2 : Number of Spots in nuclei selected- per Cell : >=3
		Boolean Operations : F1 and F2
**Select Population**	**Population :** Nuclei Selected final	**Method :** Filter by Property
Output Population : High NOTCH		F1 : Number of Spots / area cytoplasm :>=15
		F2 : Number of Spots / area Nucleus :>=3
		F3 : Number of Spots in nuclei selected- per Cell : >=10
		Boolean Operations : F1 and F2 or F3
**Modify Population**	**Population :** High Notch	**Method :** Cluster by Distance
Output Population : NOTCH clusters	**Region :** Cell	Distance : 2 µm
Output Region : NOTCH cluster region		Area : > 0 µm2
		Fill Holes
**Calculate Properties**	**Population :** NOTCH Clusters	**Method :** By Related Population
Output Properties : N of High NOTCH per Cluster		Related Population : High Notch
		Number of High Notch
**Select Population**	**Population :** NOTCH Clusters	**Method :** Filter by Property
Output Population : NOTCH Clusters (>3/cluster)		High NOTCH per Cluster : >= 3

**Define Results**		
**Method :** List of Outputs		**Method :** Formula Output
**Population : Nuclei Selected final**		Formula : a/b
Number of Objects		Variable A : Number of Junctions final - Number of Objects
Cell morphology Area [µm2]	Mean per well	Variable B : Nuclei - Number of Objects
Cell morphology Roundness	Mean per well	Output Name : Jn (% n Junctions final/n nuclei)
Cell morphology Width [µm]	Mean per well	
Cell morphology Length [µm]	Mean per well	**Method :** Formula Output
Cell morphology Ratio Width to Length	Mean per well	Formula : a/b*100
Nuclei morphology Area [µm2]	Mean per well	Population Type : Objects
Nuclei morphology Roundness	Mean per well	Variable A : [-/-] Cells having no spots - Number of Objects
Nuclei morphology Width [µm]	Mean per well	Variable B : Nuclei Selected final - Number of Objects
Nuclei morphology Length [µm]	Mean per well	Output Name : % n-/-
Nuclei morphology Ratio Width to Length	Mean per well	
STAR Properties (Cell Morphology STAR)	Mean per well	
**Population : [-/-] Cells having no spots**		**Method :** Formula Output
Number of Objects		Formula : a/b*100
**Population : [+/-] Cells having spots only in cytoplasm**		Population Type : Objects
Number of Objects		Variable A : [+/-] Cells having spots only
**Population : [+/+] Cells having spots in both cytoplasm & nulcei**		in cytoplasm (no nuclei spots) - Number of Objects
Number of Objects		Variable B : Nuclei Selected final - Number of Objects
		Output Name : % n+/-
		**Method :** Formula Output
		Formula : a/b*100
		Population Type : Objects
		Variable A : [+/+] Cells having spots in both
		cytoplasm & nulcei - Number of Objects
		Variable B : Nuclei Selected final - Number of Objects
		Output Name : % n+/-

Table S2: High content pipeline extracted from Columbus, colours correspond to Figure 1C and S2. Each step is detailed to allow replication in other software.
